# Platinum and Iridium Oxide Co-modified TiO_2_ Nanotubes Array Based Photoelectrochemical Sensors for Glutathione

**DOI:** 10.3390/nano10030522

**Published:** 2020-03-13

**Authors:** Jing Tian, Peng Zhao, Shasha Zhang, Guona Huo, Zhaochen Suo, Zhao Yue, Shoumin Zhang, Weiping Huang, Baolin Zhu

**Affiliations:** 1 College of Chemistry, The Key Laboratory of Advanced Energy Materials Chemistry (Ministry of Education), Tianjin Key Lab of Metal and Molecule-based Material Chemistry, Nankai University, Tianjin 300071, China; tianjingnk@nankai.edu.cn (J.T.); zhaopeng@nankai.edu.cn (P.Z.); 2120170933@mail.nankai.edu.cn (S.Z.); 15230153709@163.com (G.H.); zc.suo@mail.nankai.edu.cn (Z.S.); zhangsm@nankai.edu.cn (S.Z.); hwp914@nankai.edu.cn (W.H.); 2 Department of Microelectronics, Nankai University, Tianjin 300350, China; 3 National Demonstration Center for Experimental Chemistry Education (Nankai University), Tianjin 300071, China

**Keywords:** TiO_2_ nanotubes array/Ti electrode, Pt, IrO_2_, co-modification, photoelectrochemical sensor, glutathione

## Abstract

Oriented TiO_2_ nanotubes, which are fabricated by anodic oxidation method, are prospective in photoelectrochemical analysis and sensors. In this work, Pt and IrO_2_ co-modified TiO_2_ nanotubes array was prepared via a two-step deposition process involving the photoreductive deposition of Pt and chemical deposition of IrO_2_ on the oriented TiO_2_ nanotubes. Due to the improved separation of photo-generated electrons and holes, Pt-IrO_2_ co-modified TiO_2_ nanotubes presented significantly higher PEC activity than pure TiO_2_ nanotubes or mono-modified TiO_2_ nanotubes. The PEC sensitivity of Pt-IrO_2_ co-modified TiO_2_ nanotubes for glutathione was also monitored and good sensitivity was observed.

## 1. Introduction

Photoelectrochemical (PEC) detection is a rapidly developing technique, due to its characteristics of high sensitivity, fast response speed, and simple instrumentation [1,2,3,4,5,6], and attracts a lot of research attention for detection of biomolecules, such as NADH, glucose, and glutathione, which are closely related to many serious diseases [7]. In PEC biosensors, photocurrent is produced by the physical and chemical interactions between biomolecules; photoactive species are identified as detection signal. The relationship between the biomolecule concentrations and photocurrent provides the foundation for PEC sensors. This promising analytical technology relies intensively on photoelectrodes. Hence, the selection of a proper photoelectrode is critical.

Diverse nanomaterials, such as Au [8], ZnO [9,10], CdS [11,12], TiO_2_ [13,14], and porphyrin [15,16], have been exploited as electrodes. Among them, TiO_2_ nanomaterials have been widely investigated due to their high PEC activity, stable performance, good biological compatibility, and obvious surface effect [17,18,19]. TiO_2_ nanotube arrays can be grown directly on Ti substrates (TiO_2_NTs/Ti) by anodic oxidation, which are good candidates for PEC electrodes. For such electrodes, Ti substrates have superior performance of electronic conduction, stability, and exhibit excellent compatibility, which enable them to be excellent implantable devices [20]. In addition, the unique one-dimensional nanostructure of TiO_2_ nanotube arrays accelerates electron transport and shortens the transfer distance of photogenerated carriers [21]. The tubular structure of TiO_2_NTs can also facilitate the high dispersion of modified components and adsorption of aimed biomolecules. However, the application of TiO_2_ in PEC detection is limited by its wide band gap and fast recombination of photogenerated charges, which can lower the photoenergy conversion efficiency and sensitivity of PEC sensors. A typical method to solve these problems is modification the TiO_2_ photoelectrode with noble metals [22,23,24] or metal oxides [25,26]. The presence of noble metal results in the formation of a Schottky barrier at the metal-semiconductor interface, which can reduce the recombination of photogenerated charges and promote the separation of photogenerated charge carriers [27,28,29]. For instance, He’s group prepared Pt particles modified TiO_2_ film, and the photocurrent of 3 wt% Pt modified TiO_2_ film under UV irradiation was 1.5 times higher than that of bare TiO_2_ film [30]. In contrast to Pt, IrO_2_ can intercept photogenerated holes from semiconductors and mediate the hole transfer process [31,32]. The synergistic effect of Pt and IrO_2_ has been reported by Yuan’s group [33]. Pt functioned as an electron collector while IrO_2_ functioned as a hole capture, which may have led to significant charge separation and increased photocatalytic activity. 

**Scheme 1:**
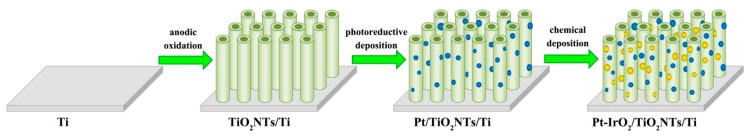
Schematic representation of preparation process of Pt-IrO_2_/TiO_2_NTs/Ti electrode.

Glutathione (γ-glutamyl-cysteinyl-glycine, GSH), an important tripeptide, plays a major role in many biological functions such as gene expression regulation, cell protection, immune regulation, enzyme activity, and metabolic regulation, etc. [34,35]. Cellular concentration of GSH is related to a variety of human diseases [34,36], and the detection of GSH is urgent because of its importance in physiological circumstances. In this paper, Pt and IrO_2_ nanoparticles were loaded on TiO_2_NTs/Ti electrodes to form a Pt-IrO_2_/TiO_2_NTs/Ti electrode as shown in Scheme 1, leading to a novel PEC biosensing platform, which was then applied for the biodetection of GSH. This biosensor showed good sensing performance of GSH with a rapid response. The functions of Pt and IrO_2_ in this system were also explored.

## 2. Experimental Section

### 2.1. Reagent and Apparatus

All chemical reagents were analytical grade and used without further purification. Reduced glutathione (GSH), PBS (0.1 M phosphate buffer, pH = 7.4 at 25 °C), H_2_IrCl_6_·xH_2_O and Ti foil were purchased from Sigma (Merck Life Science (Shanghai) Co., Ltd. Shanghai, China). H_2_PtCl_6_·4H_2_O (Keruisi Chemical Reagent Co., Ltd. Tianjin, China) was used as platinum precursor. All aqueous solutions were prepared with 18 MΩ ultra purified water.

The chemical state of the elements on the photoelectrode was determined by Axis Ultra DLD multi-technique X-ray photoelectron spectrometer (Kratos Analytical Ltd., Manchester, UK). The phase structure of the catalysts was characterized by Rigaku D/Max-2500 X-ray diffractometer with Cu Kα radiation (Rigaku, Tokyo, Japan). Transmission electron microscopy (TEM) was performed using a Talos F200X G2 transmission electron microscope (FEI, Waltham, MA, USA). Scanning electron microscopy (SEM) was performed using a JSM-7500F scanning electron microscope (JEOL, Tokyo, Japan).

### 2.2. Preparation of TiO_2_NTs/Ti

According to the reported anodic oxidation method [37,38], TiO_2_ nanotubes arrays were prepared on Ti foil (30 mm × 10 mm × 0.127 mm). First, Ti foils were cleaned by sonicating in acetone, alcohol, and deionized water for 15 min, respectively. Then they were etched in mixture liquor HF: HNO_3_: H_2_O = 1:4:5 for 30 s to remove the oxide layer. The prepared Ti foils were anodized by anodic inoxidation at 30 V for 6 h in an electrolytic solution (0.3 wt% NH_4_F +2 vol% H_2_O in ethylene glycol) under magnetic stirring at room temperature. The samples were sonicated for 15 min in ethanol and then dried in air. Finally, the as-prepared samples were calcinated at 400 °C for 2 h in a muffle furnace to obtain TiO_2_NTs/Ti.

### 2.3. Preparation of Pt-IrO_2_/TiO_2_NTs/Ti

Pt-IrO_2_/TiO_2_NTs/Ti was prepared via a two-step deposition process involving the photoreductive deposition of Pt and chemical deposition of IrO_2_ on TiO_2_NTs/Ti. First, TiO_2_NTs/Ti was dipped into a 0.05 mM H_2_PtCl_6_ solution (ethanol and deionized water with volume ratio 1:1) under the irradiation of 300 W UV light for 3 h to get the Pt modified TiO_2_ nanotube array electrode (Pt/TiO_2_NTs/Ti). Then, the Pt/TiO_2_NTs/Ti was dipped into a 0.5 mM H_2_IrCl_6_ aqueous solution for 10 min, and dried in the oven at 150 °C for 10 min. After repeating the immersion process 5 times under the same conditions, the as-obtained sample was further annealed at 400 °C for 2 h to obtain Pt-IrO_2_/TiO_2_NTs/Ti. For comparation, IrO_2_ modified TiO_2_NTs (IrO_2_/TiO_2_NTs/Ti) were prepared by using TiO_2_NTs/Ti as support.

### 2.4. Photoelectrochemical Measurement System

The photoelectrochemical experiments were performed with a CHI 604D electrochemical analyzer (CH Instruments, USA) using a three-electrode system. In the system, Ag/AgCl in 3 M KCl, platinum wire, and prepared electrodes were used as the reference electrode, counter electrode, and working electrode, respectively. An LED light source (M365L2: 365 nm, 90 mW) was fixed 30 cm above the working electrode and modulated by Transistor-Transistor Logic (TTL) out from a SR830 lock-in amplifier. PEC detection was carried out in PBS (pH = 7.4) containing different concentrations of GSH at room temperature. The switching on and off of the light source was controlled manually.

## 3. Results and Discussions

### 3.1. Microstructure Analysis

**Figure 1:**
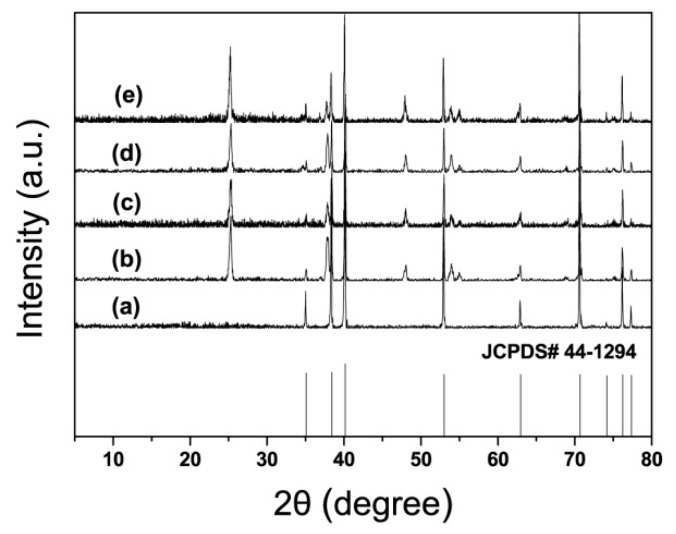
XRD patterns of (**a**) Ti foil; (**b**) TiO_2_NTs/Ti; (**c**) Pt/TiO_2_NTs/Ti; (**d**) IrO_2_/TiO_2_NTs/Ti; (**e**) Pt-IrO_2_/TiO_2_NTs/Ti.

The XRD patterns of Ti foil, bare TiO_2_NTs, and the modified TiO_2_NTs/Ti are shown in Figure 1. The characteristic peaks of Ti (JCPDS# 44-1294) emerged in all patterns, because Ti foils were substrates. The peaks appearing at 25.28°, 37.80°, 48.05°, 53.89°, 55.06°, 62.69° observed in Figure 1b–e correspond to the diffraction of (101), (004), (200), (105), (211), (204) crystal planes of anatase TiO_2_ (JCPDS# 21-1272), respectively. However, diffraction peaks of Pt or IrO_2_ were not observed in the modified electrodes, which should be attributed to their high dispersion and low content. 

**Figure 2:**
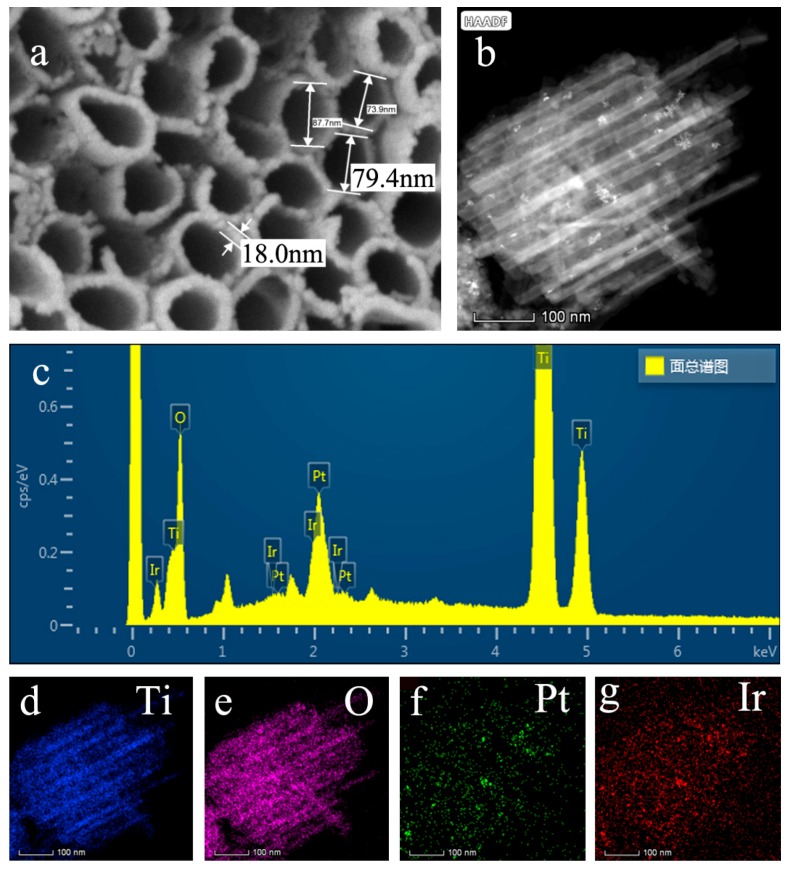
(**a**) Top-view SEM of TiO_2_NTs; (**b**) The TEM image of Pt-IrO_2_/TiO_2_NTs/Ti; (**c**) The EDS of Pt-IrO_2_/TiO_2_NTs/Ti; (**d**–**g**) The EDS mappings of Pt-IrO_2_/TiO_2_NTs/Ti.

The morphology and microstructure of the as-prepared TiO_2_NTs/Ti was characterized by SEM and the Pt-IrO_2_/TiO_2_NTs/Ti was characterized by TEM. The top view SEM image (Figure 2a) shows that the bare TiO_2_ nanotubes were highly ordered, with an average diameter of 80 nm and wall thickness of 18 nm. It is also clearly observed that the tubes were open on the top. 

Figure 2b shows the microstructure of TiO_2_NTs modified with Pt and IrO_2_. To confirm the presence of Pt and IrO_2_ on the TiO_2_NTs, EDS and elemental mapping were carried out. The EDS (Figure 2c) showed that atomic percentages of Pt and Ir in Pt-IrO_2_/TiO_2_NTs/Ti were 0.70% and 0.41%, respectively. The elemental mappings present the existence of Pt and IrO_2_ visually, as shown in Figure 2d–g. It can observe that the distributed Pt and Ir elements were homogeneous.

**Figure 3:**
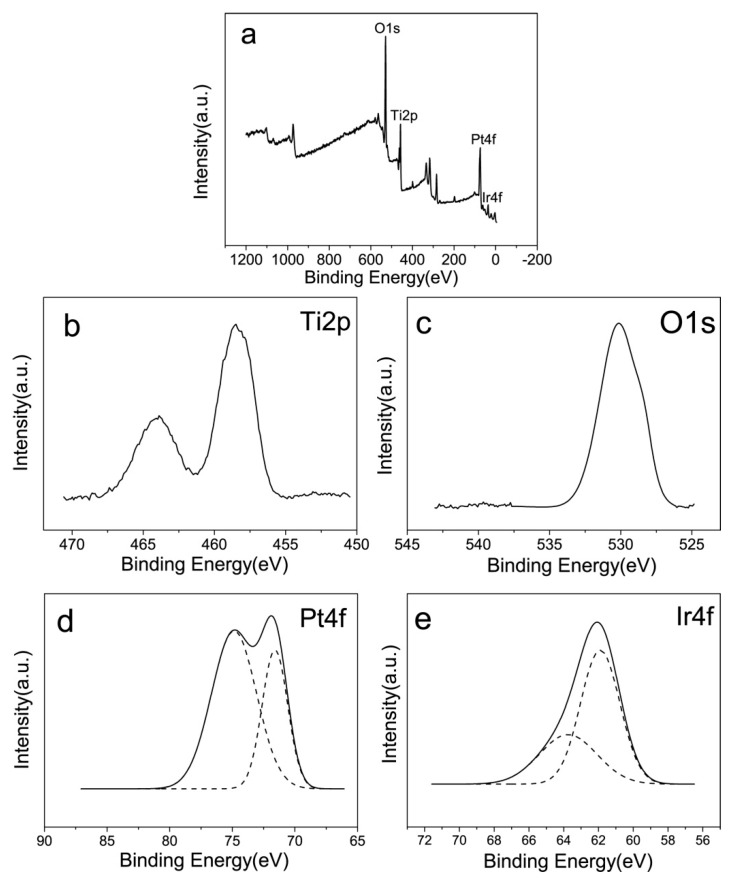
(**a**) XPS spectra of Pt-IrO_2_/TiO_2_NTs/Ti, (**b**) Ti2p, (**c**) O1s, (**d**) Pt4f; (**e**) Ir4f.

To further investigate the composition and elemental valences of the modified electrode, XPS analysis was performed. Figure 3 shows the XPS spectrum of Pt-IrO_2_/TiO_2_NTs/Ti, and corresponding high-resolution spectra of Ti2p, O1s, Pt4f, and Ir4f (Figure 3b–e). Figure 3a shows that the sample contained Ti, O, Pt and Ir, indicating successful deposition of Pt and IrO_2_ on the TiO_2_NTs, consistent with the EDS results. In Figure 3b, two peaks at BE of 464.0 eV and 458.5 eV in Pt-IrO_2_/TiO_2_NTs/Ti were assigned to Ti2p_1/2_ and Ti2p_3/2_ respectively, indicating the existence of Ti^4+^ in TiO_2_ [14,39,40]. In Figure 3c, the peak at BE of 529.5 eV was assigned to Ti-O [41].

As displayed in Figure 3d, the satellite peaks at 75.0 eV and 71.6 eV are strong evidence for Pt4f_5/2_ and Pt4f_7/2_, which confirms the formation of metallic Pt after the photoreduction deposition process [42,43]. After deconvolution, the high-resolution Ir4f (Figure 3d) showed the peak at 64.9 eV for Ir4f_5/2_ and 62.0 eV for Ir4f_7/2_, which is the characteristic of Ir^4+^ in IrO_2_ [41,44,45]. Consequently, Pt and IrO_2_ were successfully deposited on the TiO_2_NTs/Ti electrode, and Pt-IrO_2_ co-modified electrode was fabricated.

### 3.2. Photoelectrochemical Performance

**Figure 4:**
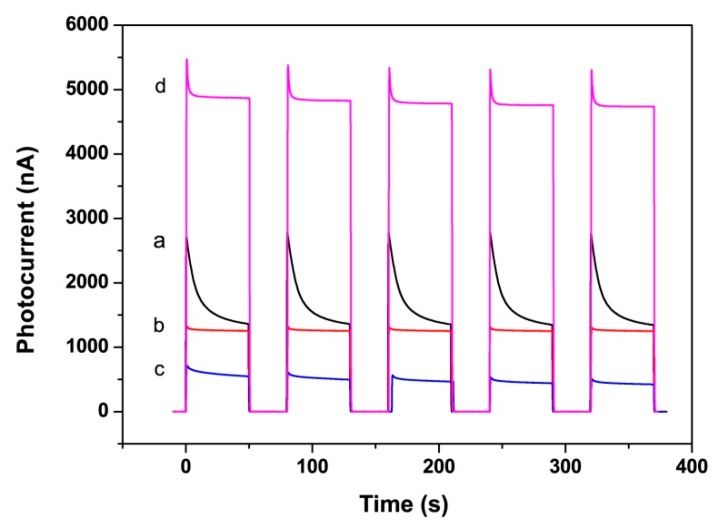
Photocurrent responses of different electrodes: (**a**) TiO_2_NTs/Ti, (**b**) Pt/TiO_2_NTs/Ti, (**c**) IrO_2_/TiO_2_NTs/Ti, (**d**) Pt-IrO_2_/TiO_2_NTs/Ti electrodes in 0.1M PBS solution under 365 nm LED irradiation.

A photocurrent-time curve can be used to characterize the separation effect of photogenerated carriers, and the increase of photocurrent indicates the low recombination probability of photogenerated electron-holes. Figure 4 shows the photocurrent-time curves of different electrodes in 0.1 M PBS solution under 365 nm LED UV light irradiation. From Figure 4a, it can be seen that the photocurrent curve of the bare TiO_2_NTs electrode had an anodic photocurrent spike at the initial time of irradiation, and then continuously decreased until a constant current was reached. However, the decorated TiO_2_NTs electrodes could generate stable photocurrent more rapidly under the same condition, as shown in Figure 4b–d.

Under illumination, TiO_2_ could absorb UV light and generate electron-hole pairs, and electrons from the TiO_2_ electrode could transfer to Pt. So the surface of Pt nanoparticles appeared negatively charged and TiO_2_ appeared positively charged. The Schottky barrier prevents electrons from recombining with holes, and promotes the migration of charges, thus improves the stability of the photocurrent (Figure 4b). For IrO_2_/TiO_2_NTs/Ti, the photoinduced holes were taken by IrO_2_, effectively preventing the combination of electron-hole. So the photocurrent of IrO_2_/TiO_2_NTs/Ti electrode also could quickly stabilize, as shown in Figure 4c. In the Pt-IrO_2_ modified TiO_2_ system, due to the synergistic effect of Pt and IrO_2,_ the recombination of photogenerated electrons and holes could be effectively blocked. The Pt-IrO_2_/TiO_2_NTs/Ti electrode in Figure 4d exhibited an apparent and stable photocurrent signal compared with the TiO_2_NTs/Ti.

However, the mono Pt- or IrO_2-_modified TiO_2_NTs/Ti exhibited lower photocurrent responses than bare TiO_2_NTs/Ti. The reason may be that Pt- and IrO_2-_loading on TiO_2_NTs shelter the TiO_2_NTs from the light illumination, which could reduce the efficiency of photogenerated carriers. Another reason may be that when Pt or IrO_2_ nanoparticles appeared alone on the TiO_2_, both photogenerated electrons and holes may transfer to Pt or IrO_2_ nanoparticles, making them act as recombination centers [46,47]. However, if Pt and IrO_2_ nanoparticles co-exist, electrons would move to Pt, while holes move to IrO_2_. The electron-hole recombination would be suppressed, leading to a higher photocurrent.

### 3.3. PEC biosensing Application for GSH

**Figure 5:**
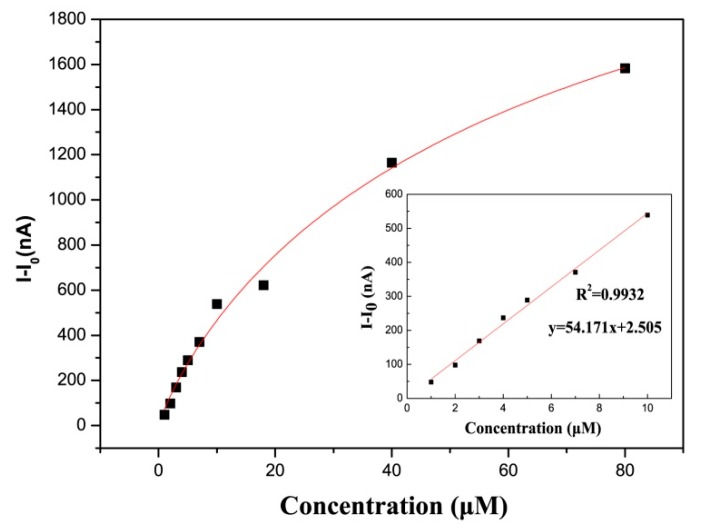
The curve of Pt-IrO_2_/TiO_2_NTs/Ti for the detection of different concentrations for GSH.

On the basis of photoelectrochemical performance, the application of the fabricated Pt-IrO_2_/TiO_2_NTs/Ti for detection of GSH was investigated. The electrode was kept in PBS at +0.3 V bias voltage under UV illumination for 5 min as pretreatment, and then the positive photocurrent was detected in the presence of GSH in PBS. As shown in Figure 5, the photocurrent increased with the addition of GSH under illumination, fitted with the Langmuir curve. The inset presented in Figure 5 shows that the photocurrent response of the Pt-IrO_2_/TiO_2_NTs/Ti biosensor was proportional to GSH concentration in the range of 1~10 μM with the regression equation of I-I_0_(μA) = 2.505 + 54.171C_GSH_(uM) (R^2^ = 0.9932) (I represents the photocurrent obtained in the presence of GSH, and I_0_ is the blank photocurrent). The detection limit (LOD) of the sensor can be obtained from the formula LOD = 3 Sb/S (Sb = standard deviation of blank signal, S = sensitivity), and the calculated value was 0.8 μM.

**Scheme 2:**
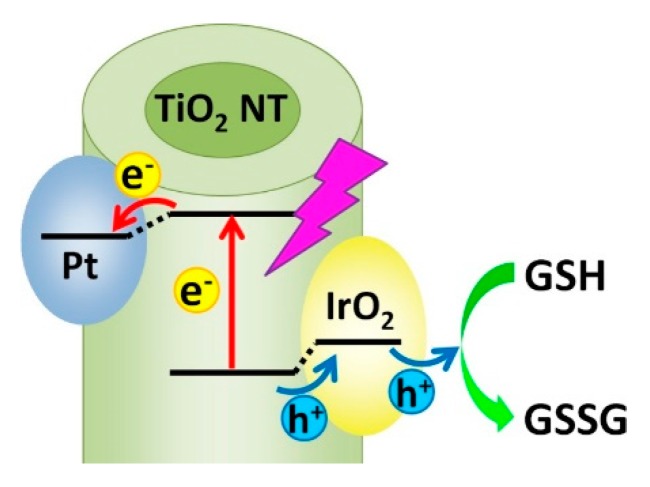
Schematic illustration of the PEC process for GSH detection by Pt-IrO_2_/TiO_2_NTs/Ti biosensor.

Scheme 2 illustrates the PEC process for GSH detection by Pt-IrO_2_/TiO_2_NTs/Ti biosensor. Under the illumination of UV light, electron-hole pairs were generated in TiO_2_. Due to the Schottky barrier at the Pt/TiO_2_ interface, electrons moved from TiO_2_ electrode to Pt. Simultaneously, the photoinduced holes were taken by IrO_2_, and then oxidized GSH to GSSG.

Table 1 summarizes the comparison of analytical performance of various GSH biosensors. The Pt-IrO_2_/TiO_2_NTs/Ti biosensor had a relatively reasonable linear range in 1-10 μM. Compared with other non-enzymatic sensors, Pt-IrO_2_/TiO_2_NTs/Ti was sensitive and could be used at relatively low GSH concentrations. Though enzymatic sensors offer high sensitivity, non-enzymatic sensors are widely recognized for their good advantages of simple operation, lack of need for expensive equipment, and high stability. The stability of the Pt-IrO_2_/TiO_2_NTs biosensor was tested by measuring photocurrent response after 30 days (see Supplementary Figure S1). The photocurrent did not decrease significantly, indicating that the electrode’s good stability for GSH detection. This should contribute to the good stability of Pt and IrO_2_ on TiO_2_NTs/Ti electrodes. 

In summary, a PEC sensor for GSH was designed using Pt-IrO_2_/TiO_2_NTs/Ti as an electrode. Pt and IrO_2_ were evenly distributed on TiO_2_NTs/Ti. Due to the synergistic effects of Pt and IrO_2,_ the Pt-IrO_2_/TiO_2_NTs/Ti electrode exhibited an apparent and stable photocurrent signal compared with the TiO_2_NTs/Ti or the mono-modified ones. The photocurrent signals of the Pt-IrO_2_/TiO_2_NT/Ti biosensor was linear to GSH concentration in the range of 1~10 μM. Other kinds of modified TiO_2_NTs/Ti electrodes which may exhibit high PEC sensitivity under visible light are anticipated.

## Figures and Tables

**Table 1:** Comparison of various GSH sensors.

	GSH Biosensor	Linear Range (μM)	Detection Limit (μM)	Ref.
Non-enzymatic sensor	Pt-IrO_2_/TiO_2_NTs/Ti	1–10	0.8	This work
	Cu_2_O/ZnO	1–10 and 20–100	0.8	[48]
	GR-CdS/ITO	10–1500	3	[49]
	rGO/ZnO	10–200	2.17	[50]
	Porphyrin-Functionalized TiO_2_-ITO	50–2400	30	[16]
Enzymatic sensor	IrO_2_-Hemin-TiO_2_ nanowire arrays	0.01–10	0.01	[17]

## Supplementary Materials

The following are available online at https://www.mdpi.com/2079-4991/10/3/522/s1, Figure S1: The curve of Pt-IrO_2_/TiO_2_NTs/Ti (after 30 days) for the detection of different concentrations for GSH.
