# Supplementary material for: Platinum and Iridium Oxide Co-modified TiO2 Nanotubes Array Based Photoelectrochemical Sensors for Glutathione
Source: Nanomaterials (Basel). 2020 Mar 13;10(3):522. doi: 10.3390/nano10030522 (PMC7153253; doi:10.3390/nano10030522)
Supplement: Supplementary file 1 [file nanomaterials-10-00522-s001.pdf]

Platinum and Iridium Oxide Co-modified TiO_2 Nanotubes Array Based Photoelectrochemical Sensors for Glutathione

Jing Tian[1], Peng Zhao[1], Shasha Zhang[1], Guona Huo[1], Zhaochen Suo[1], Zhao Yue[2,*], Shoumin Zhang[1], Weiping Huang[1] and Baolin Zhu[1,3,*]

[1] College of Chemistry, The Key Laboratory of Advanced Energy Materials Chemistry (Ministry of Education), Tianjin Key Lab of Metal and Molecule-based Material Chemistry, Nankai University, Tianjin 300071, China; tianjingnk@nankai.edu.cn (J.T.); zhaopeng@nankai.edu.cn (P.Z.); 2120170933@mail.nankai.edu.cn (S.Z.); 15230153709@163.com (G.H.); zc.suo@mail.nankai.edu.cn (Z.S.); zhangsm@nankai.edu.cn (S.Z.); hwp914@nankai.edu.cn (W.H.)

[2] Department of Microelectronics, Nankai University, Tianjin 300350, China

[3] National Demonstration Center for Experimental Chemistry Education (Nankai University), Tianjin 300071, China

* Correspondence: yuezhao@nankai.edu.cn (Z.Y.); zhubaolin@nankai.edu.cn (B.Z.)

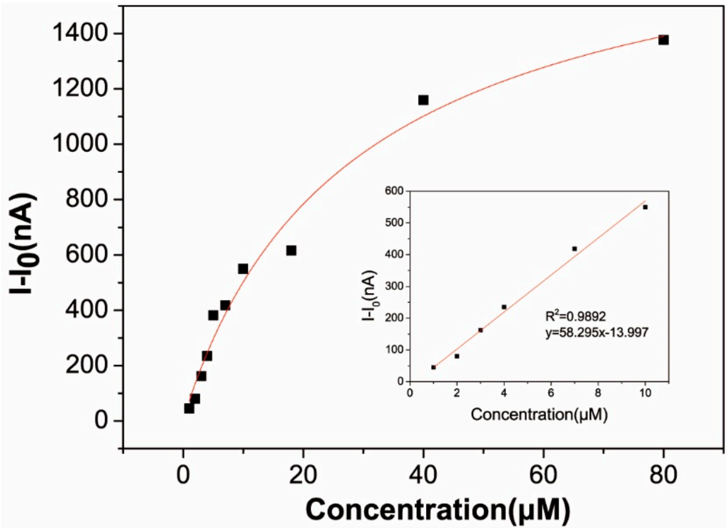

Figure S1. The curve of Pt-IrO_2/TiO_2NTs/Ti (after 30 days) for the detection of different concentrations for GSH.
